# Application Value of Magnetic Resonance Arthrography of the Shoulder in Nursing and Diagnosis of Patients with Shoulder Joint Injury

**DOI:** 10.1155/2021/3051578

**Published:** 2021-08-23

**Authors:** Si Chen, Yuanyuan Shi, Pan Xue, Xue Chen

**Affiliations:** ^1^Geriatric Medicine, The Second Hospital of Jilin University, Changchun 130041, China; ^2^Department of Nursing, The Second Hospital of Jilin University, Changchun 130041, China; ^3^Department of Orthopedics, The Second Hospital of Jilin University, Changchun 130041, China

## Abstract

Supraspinatus tendon injury is a common clinical shoulder joint disease and is one of the most common causes of shoulder pain and dysfunction. Supraspinatus tendon injury will lead to articular cartilage injury and degeneration, then cause joint disease, seriously affect the quality of life of patients, and bring a huge burden to the family and society. This paper mainly studies and evaluates the application value of special signs of shoulder joint and indirect MR imaging in the diagnosis of supraspinatus tendon injury. Through a series of special examinations for the diagnosis of supraspinatus tendon injury in 90 patients, including zero degree abduction resistance test, arm drop test, Jobe test, Neer sign, and Hawkins sign, all patients in the study underwent indirect magnetic resonance imaging of the shoulder joint. Finally, arthroscopic examination results were used as the “gold standard” to evaluate and analyze the diagnosis. The results showed that among the special signs, the specificity of the falling-arm test was the highest (72.2%) in the diagnosis of full-thickness supraspinatus tendon injury. Hawkins sign had the highest sensitivity (84.0%). In the diagnosis of partial supraspinatus tendon injury, the specificity of the Jobe test was the highest, which was 66.6%. The Neer sign had the highest sensitivity of 50.0%. In the diagnosis of full-thickness supraspinatus tendon injury, there was no significant difference in sensitivity between indirect MRI and Hawkins sign, but the diagnostic specificity of indirect MRI was higher than that of special sign examination. In the diagnosis of partial supraspinatus tendon injury, the sensitivity and specificity of indirect MR imaging are higher than those of special sign examination.

## 1. Introduction

Rotator cuff injury has attracted much attention since Smith first put forward the concept of rotator cuff injury in 1834. These concerns are not only due to the research interest of scholars, but also due to the needs of a large number of patients. The study found that the incidence of partial or full-thickness rotator cuff injury increased significantly after age 50, exceeding 50 percent in people over 70 and more than 80 percent in people over 80. What deserves more attention is the progression of the disease after the injury of the rotator cuff. Yamanaka et al. observed 40 patients with partial rotator cuff injury without surgical treatment. After more than one year of follow‐up, changes of rotator cuff injury is examined with shoulder arthrography. The results showed that only 4 patients had obvious healing of injured rotator cuff. The extent of injury was reduced in four patients. At the same time, 21 patients had an expanded rotator cuff lesion, and 11 patients shifted from a partial rotator cuff lesion to a full-thickness lesion.

Among the tendons that make up the rotator cuff, the supraspinatus tendon is located at the top of the rotator cuff. Compared with other tendons, the supraspinatus tendon is most vulnerable to the extrusion of the acromial peak and the coracoacromial arch and is the tendon with the highest incidence of degeneration and injury. Because of the high incidence of rotator cuff injury and the exacerbation of the course of disease, it is of great significance to improve the understanding of rotator cuff injury, especially supraspinatus tendon injury. The previous concept of “periarthritis of the shoulder” is obviously too broad and general, and the early diagnosis of supraspinatus tendon injury is essential to prevent further progression of the disease. However, the clinical symptoms of supraspinatus tendon injury are diverse, and there is a lack of special clinical symptoms for diagnosis. Routine physical examination often fails to provide sufficient diagnostic reference information. Therefore, various specific examinations for special signs have emerged as the times require. At the same time, a variety of imaging examinations also provide useful diagnostic information from different perspectives, and how to choose efficient and reliable diagnostic techniques to help the clinical screening and diagnosis of supraspinatus tendon injury has become one of the hot spots of attention and research.

Physical examination was performed to assess supraspinatus tendon injury by an impingement test that elicits pain and by examining the strength of the supraspinatus muscle, and the diagnosis of supraspinatus tendon injury by indirect magnetic resonance arthrography was performed.

Imaging examination is an essential and important means for the diagnosis of supraspinatus tendon injury, and it is also helpful to determine the degree and type of supraspinatus tendon injury and whether there are other accompanying shoulder diseases. There is no doubt that imaging examination plays an important role in the diagnosis, treatment, and prognosis evaluation of supraspinatus tendon injury. Imaging examinations for supraspinatus tendon injuries mainly include the following types: X-ray examination (X-ray examination cannot directly observe the rotator cuff and other soft tissues, mainly through some indirect signs to determine whether there is rotator cuff injury), CT examination (CT examination can clearly show the shoulder joint fault structure and three-dimensional reconstruction), ultrasound, which uses a range of physical properties of ultrasound to identify and image soft tissue, and MRI, which is a great advance in the imaging diagnosis of rotator cuff. The basic element of the imaging principle of MRI is the hydrogen nuclei contained in the tissues of the body. Currently, the diagnosis of the supraspinatus tendon injury mainly includes plain MRI, direct MRI arthrography, and indirect MRI arthrography.

The research of MR arthrography in the examination of shoulder joint injury is developing continuously. MR arthrography (MRA) is often used to evaluate intra-articular disorders of the shoulder. Liao et al. discussed the diagnostic value of MR arthrography for rotator cuff injury [1]. Zhang et al. discussed the diagnostic value of conventional MRI and MR arthrography in flapping injury of shoulder joint [2]. Xiao et al. discussed the diagnostic value of magnetic resonance arthrography of the shoulder for partial rotator cuff tear [3]. Nassef et al. proved that MR arthrography is the preferred imaging examination method for patients with shoulder instability [4]. Alaia et al. discussed the standard MR imaging and arthrography protocols routinely used in clinical practice, as well as more innovative sequence and reconstruction techniques facilitated by the increase of high field intensity magnets and multichannel phased array surface coils and the combination of artificial intelligence [5].

Studies are also under way to combine MRI arthrography with other procedures. Pan et al. summarized the diagnosis and measurement methods of bone defect in anterior shoulder instability (scapular glenoid defect and Hill–Sachs lesion): X‐ray, CT, MRI, arthroscopy, and arthrography are common methods for the diagnosis of humeral head anterior glenoid defect and Hill–Sachs lesion [6]. Foti et al. compared the diagnostic accuracy of dual‐energy CT arthrography (DE‐CTA) and magnetic resonance arthrography (MRA) in describing labial lacerations [7]. Fu et al.'s objective was to explore the application of CT combined with MRI in the diagnosis of shoulder injury and to provide reference for clinical application [8]. Bucha et al. studied the value of shoulder magnetic resonance in the diagnosis of shoulder instability and compared it with arthroscopy [9].

This paper mainly studies and evaluates the application value of special signs of shoulder joint and indirect MR imaging in the diagnosis of supraspinatus tendon injury.

## 2. Research Methods

### 2.1. Research Object

A total of 90 cases were enrolled, including 46 males and 44 females, aged 42 to 75, with an average age of 58.6 years (48 cases of right shoulder and 42 cases of left shoulder and duration ranging from 4 to 24 months, with an average of 8.5 months). Eighteen patients had a history of shoulder trauma, and 12 patients complained of shoulder strain. The other cases had no obvious cause of disease. There were 10 cases of coronary heart disease, 12 cases of diabetes, and 19 cases of hypertension in this group. There were 52 cases of dominant limb and 38 cases of nondominant limb. Visual analog scale of pain (VAS score), shoulder function score (UCLA score, Constant score, and ASE score), and range of motion of shoulder were measured preoperatively in all patients.

### 2.2. Examination of Special Physical Signs

The examination of special physical signs was performed and recorded by an attending physician with more than 5 years of experience in sports medicine and a doctoral candidate in sports medicine. All the patients were examined on both sides of the shoulder. The order of examination was first the physical examination of the walking side of the shoulder and then the physical examination of the affected side of the shoulder. The contents of special physical examination are mainly for the muscle strength examination of the supraspinatus muscle and the impact test to induce shoulder pain. In order to avoid the interference of pain on the muscle strength examination results as far as possible, the impact test is arranged after the muscle strength examination.

### 2.3. Indirect Magnetic Resonance Angiography

Instruments and materials used: the MRI scanner used for scanning was the Philips Ingenia 3.0 T MRI scanner (Netherlands). The contrast agent used for indirect imaging was gadolinium spray acid meglumine gadolinate (trade name: Magenweisen (Germany); chemical name: dihydro-[N, N-bis [2-{bis (carboxymethyl) amino} ethyl] glycoyl (5-)] gadolinic acid (2-) combined with 1-deoxy-1-(methylamino)-D-glucosol (1 : 2); molecular weight: 938.00). Accessories: meglumine and water for injection.

### 2.4. Shoulder Arthroscopy

Arthroscopic personnel and equipment: all arthroscopes are performed by a team of a chief physician in sports medicine and an attending physician. Arthroscopic instruments and lenses: Smith & Nephew arthroscopic system equipment was used.

### 2.5. Statistical Methods

The diagnosis results under shoulder arthroscopy are regarded as the “gold standard” of the final diagnostic standard of all cases. Special signs of shoulder and indirect magnetic resonance angiography were used as screening diagnostic methods to be evaluated, and the results of shoulder arthroscopy were compared with the “gold standard” diagnostic results and statistically analyzed. SPSS19.0 statistical software package was used for data analysis, and X2 test was used for data statistical analysis. If *P* < 0.05, the difference was considered to be statistically significant.

## 3. Results

### 3.1. Examination of Special Physical Signs

Procedures for checking special physical signs are as follows:

  Abduction resistance test at zero position: the patient's two upper limbs are placed at the side of the body, and the examinator stands behind the patient's body against the patient's upper limb abduction movement. If the patient feels obvious pain or cannot resist resistance and cannot abduct the upper limbs, the test is positive.
  The dropping arm test: the examiner abducted the patient's shoulder joint to more than 90 degrees and let the patient keep it by himself. The test is positive if the patient is unable to maintain the abduction position until the upper limb falls.
  Jobe test (empty can test): The patient's shoulder joint abducted 80 to 90 degrees in coronal position and retracted 30 degrees in horizontal position. With the forearm pronated and the thumb pointed to the ground, the examiner applied downward pressure on the patient's wrist. Patients who are unable to resist resistance and experience significant pain and weakness are considered to be positive for the Jobe test.
  Neer sign: the examiner stands behind the patient, stabilizes the scapula on the examining side with one hand, and maintains the shoulder joint in internal rotation with the other hand, so that the fingertip of the thumb of the upper limb is downward, and then the shoulder joint on the examining side is flexed forward and lifted over the top. If pain is induced, Neer sign is positive.
  Hawkins sign: the examiner flexes the patient's elbow 90 degrees, with the shoulder adducted forward 90 degrees and the forearm in horizontal position. The examiner forces the affected forearm downward to produce internal rotation of the shoulder. If significant pain is induced, Hawkins sign is positive.

The examination results of special physical signs are as follows.

As can be seen from Table 1, in patients with full-thickness supraspinatus tendon injury, the sensitivity and specificity of the zero degree abduction resistance test were 48.0% and 65.0%, respectively. The sensitivity and specificity of falling arm test were 51.9% and 72.2%, respectively. The sensitivity and specificity of the Jobe test were 45.8% and 66.7%. The sensitivity and specificity of Neer sign were 54.2% and 61.9%. The sensitivity and specificity of Hawkins sign were 84.0% and 60.0%, respectively. The sensitivity and specificity of the zero degree abduction resistance test were 46.2% and 57.9% in patients with partial supraspinatus tendon injury. The sensitivity and specificity of falling arm test were 39.2% and 64.7%. The sensitivity and specificity of Jobe test were 40.7% and 66.6%. The sensitivity and specificity of Neer sign were 50.0% and 52.6%, respectively. The sensitivity and specificity of Hawkins sign were 45.8% and 57.1%, respectively.

Among the examinations of special signs, the falling arm test has the highest specificity and the highest sensitivity in the diagnosis of the full-thickness supraspinatus tendon injury. In the diagnosis of partial supraspinatus tendon injury, the Jobe test has the highest specificity and the highest sensitivity of Neer sign. The diagnostic accuracy of the full layer injury of supraspinatus tendon was higher than that of partial injury by special sign examination.

### 3.2. Indirect MRI Examination

**Figure 1:**
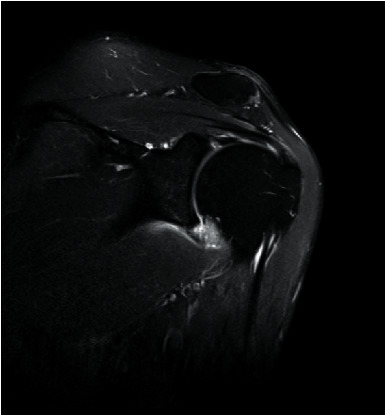
Injury of the articular portion of supraspinatus tendon 1.

**Figure 2:**
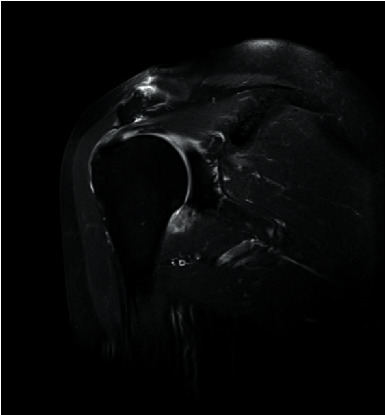
Injury of the articular part of supraspinatus tendon 2.

**Figure 3:**
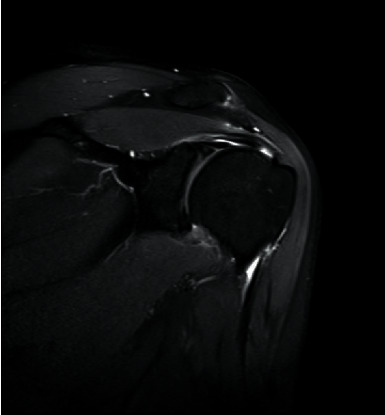
Injury of the articular part of supraspinatus tendon 3.

**Figure 4:**
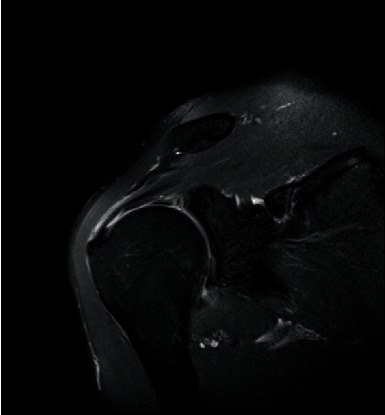
Injury of the articular part of supraspinatus tendon 4.

**Figure 5:**
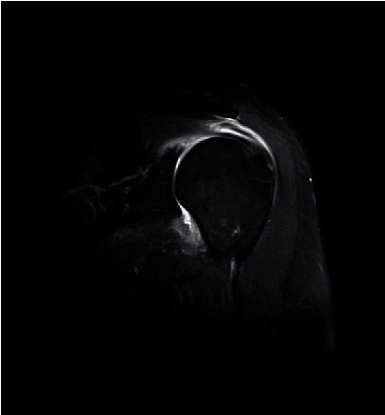
Injury of the supraspinatus tendon in tendon 1.

**Figure 6:**
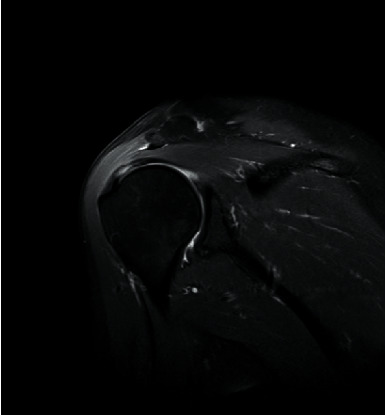
Injury of supraspinatus tendon in tendon 2.

**Figure 7:**
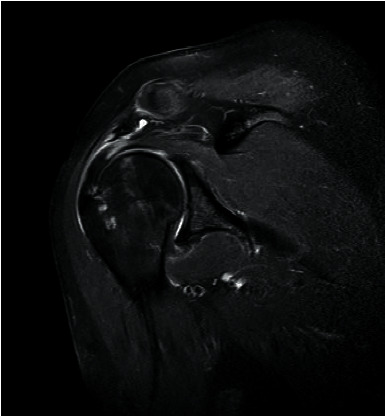
Injury of the supraspinatus tendon in tendon 3.

**Figure 8:**
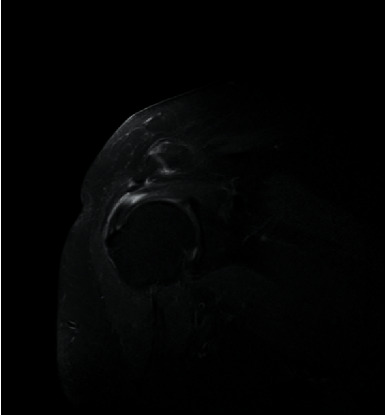
Injury of supraspinatus tendon in tendon 4.

Prior to MRI, the patient received a rapid injection of gadolinium spray diglumethamine at a dose of 0.1 mmol/kg from a peripheral vein in front of the elbow. Immediately after the injection of contrast agent, the patient was instructed to examine the lateral shoulder joint for about 15 minutes and then underwent magnetic resonance scanning. Figures 1–4 show typical cases of joint injury. During the scanning, the patient was placed in supine position, and the range value of the scanning field of view (FOV) was 160 *∗* 160 *∗* 71 mm. The scanning thickness was 3 mm, and the scanning gap was 1 mm. The precise frequency reversal recovery (SPAIR) PDW fat suppression sequence (TR 2800 ms, TE 30 ms) and matrix (Matrix) 320 *∗* 228 *∗* 18 mm were used to scan the oblique coronal plane. SPAIR T1W fat suppression sequence (TR635 ms, TE 15 ms), matrix 292 ∗ 242 ∗ 18 mm was used to scan the oblique coronal plane as well. Fast spin echo (FSE) T1WI (TR561 ms, TE15 ms) with a matrix of 320 *∗* 263 *∗* 18 mm was used for oblique sagittal scanning. For cross-sectional scanning, the SPAIR fat-inhibited PDW sequence (TR 2800 ms, TE30 ms) with a matrix of 268 *∗* 181 *∗* 18 mm was used. The MRI scan was perpendicular to the humeral shaft, extending from above the acromioclavicular joint to below the pelvis of the scapula joint. The oblique coronal scanning direction was parallel to the long axis of the supraspinatus muscle, extending from the front of the tip of the coracoid process to the rear of the scapulae. The oblique sagittal scanning direction was perpendicular to the long axis of the supraspinatus muscle and ranged from the lateral humeral head to the medial supraspinatus fossa of the shoulder joint. Figures 5–8 show a typical MRI of partial injury to the upper tendon.  Table 2 shows the results of indirect magnetic resonance angiography.

As can be seen from Table 2, the sensitivity and specificity of indirect MR imaging in the diagnosis of full-thickness injury of supraspinatus tendon were 96.7% and 93.3%, and the sensitivity and specificity of partial injury of supraspinatus tendon were 90.6% and 92.3%.

The accuracy of indirect MR imaging in the diagnosis of the full thickness injury of supraspinatus tendon was higher than that of the partial injury of supraspinatus tendon.

### 3.3. Comparison of the Results of Special Sign Examination and Indirect Magnetic Resonance Angiography

For the full-thickness injury and partial injury of supraspinatus tendon, the sensitivity and specificity of the examination with the highest sensitivity and specificity were compared with those of the indirect MRI examination (see Tables 3 and 4 for details).

In the diagnosis of full-thickness supraspinatus tendon injury, there was no significant difference between the sensitivity of MR indirect imaging and Hawkins sign, the most sensitive of the special signs (*P* > 0.05).

In the diagnosis of partial supraspinatus tendon injury, there was a significant difference between the sensitivity of MR indirect imaging and Neer sign, the most sensitive of the special signs (*P* < 0.05).

In the diagnosis of full-thickness supraspinatus tendon injury, there was a significant difference between the highest specificity of the arm test and indirect MR imaging (*P* < 0.05).

In the diagnosis of partial supraspinatus tendon injury, there was a significant difference between the specificity of Jobes test and indirect magnetic resonance imaging (*P* < 0.05).

## 4. Conclusions

1. Among the examinations of special physical signs, the falling arm test has the highest specificity and the highest sensitivity for the diagnosis of full-thickness supraspinatus tendon injury. In the diagnosis of partial supraspinatus tendon injury, the Jobe test has the highest specificity and the Neer sign has the highest sensitivity.
2. In the diagnosis of full-thickness supraspinatus tendon injury, there was no significant difference in sensitivity between indirect MR imaging and Hawkins sign, but the specificity of indirect MR imaging was higher than that of special signs.
3. In the diagnosis of partial supraspinatus tendon injury, the sensitivity and specificity of indirect MR imaging are higher than those of special sign examination.

The examination of special signs is simple and quick, which is helpful to the diagnosis of supraspinatus tendon injury and should be used as the basic diagnostic method. Indirect MR imaging is an important auxiliary diagnostic method with high sensitivity and specificity, which can be examined by multiplane images.

## Figures and Tables

**Table 1:** Results of special physical signs.

Test	Abduction resistance test		The left arm test		Jobe test		Neer		Hawkins	
Parts	Full-thickness injury	Part of the damage	Full-thickness injury	Part of the damage	Full-thickness injury	Part of the damage	Full-thickness injury	Part of the damage	Full-thickness injury	Part of the damage
True positive	12	12	14	11	11	11	13	13	21	11
A false positive	7	8	5	6	7	6	8	9	8	9
True negative	13	11	13	11	14	12	13	10	12	12
False negative	13	14	13	17	13	16	11	13	4	13
Sensitivity (%)	48	46.2	51.9	39.2	45.8	40.7	54.2	50.0	84.0	45.8
Specificity (%)	65	57.9	72.2	64.7	66.7	66.6	61.9	52.6	60.0	57.1
Positive predictive value (%)	63.2	60.0	73.7	64.7	61.1	64.7	61.9	59.1	72.4	55.0
Negative predictive value (%)	50.0	44.0	50.0	39.3	51.9	42.9	54.2	43.5	75.0	48.0
Accuracy (%)	13.0	4.1	24.1	3.9	12.5	7.3	16.1	2.6	44.0	2.9

**Table 2:** Results of indirect magnetic resonance angiography.

Parameter	Indirect magnetic resonance angiography	
Parts	Full-thickness injury	Part of the damage
True positive	29	29
A false positive	1	1
True negative	14	12
False negative	1	3
Sensitivity (%)	96.7	90.6
Specificity (%)	93.3	92.3
Positive predictive value (%)	96.7	93.5
Negative predictive value (%)	93.3	85.7
Accuracy (%)	90.0	82.9

**Table 3:** Comparison of sensitivity between indirect MRI and specific signs.

Parameter	Indirect magnetic resonance angiography	
Special signs	Full-thickness injury	Part of the damage
Pairing *X*^2^	1.273	19.361
*P* value	0.214	0.000

**Table 4:** Comparison of specificity between indirect MRI and specific signs.

Parameter	Indirect magnetic resonance angiography	
Special signs	Full-thickness injury	Part of the damage
Pairing *X*^2^	6.142	10.313
*P* value	0.033	0.023

## Data Availability

The data used to support the findings of this study are available from the corresponding author upon request.
